# Exploring the Link between Chronic Kidney Disease and Alzheimer’s Disease: A Longitudinal Follow-Up Study Using the Korean National Health Screening Cohort

**DOI:** 10.3390/biomedicines11061606

**Published:** 2023-06-01

**Authors:** Mi Jung Kwon, Young Rim Song, Joo-Hee Kim, Ji Hee Kim, Ho Suk Kang, Hyun Lim, Min-Jeong Kim, Nan Young Kim, Sangkyoon Hong, Younghee Choi, Kyueng-Whan Min, Hyo Geun Choi, Eun Soo Kim

**Affiliations:** 1 Department of Pathology, Hallym University Sacred Heart Hospital, Hallym University College of Medicine, Anyang 14068, Republic of Korea; mulank@hanmail.net; 2 Division of Nephrology, Department of Internal Medicine, Hallym University Sacred Heart Hospital, Hallym University College of Medicine, Anyang 14068, Republic of Korea; youngrim@hallym.or.kr; 3 Division of Pulmonary, Allergy, and Critical Care Medicine, Department of Medicine, Hallym University Sacred Heart Hospital, Hallym University College of Medicine, Anyang 14068, Republic of Korea; luxjhee@gmail.com; 4 Department of Neurosurgery, Hallym University Sacred Heart Hospital, Hallym University College of Medicine, Anyang 14068, Republic of Korea; kimjihee.ns@gmail.com; 5 Division of Gastroenterology, Department of Internal Medicine, Hallym University Sacred Heart Hospital, Hallym University College of Medicine, Anyang 14068, Republic of Korea; hskang76@hallym.or.kr (H.S.K.); hlim77@hallym.or.kr (H.L.); 6 Department of Radiology, Hallym University Sacred Heart Hospital, Hallym University College of Medicine, Anyang 14068, Republic of Korea; drkmj@hallym.or.kr; 7 Hallym Institute of Translational Genomics and Bioinformatics, Hallym University Medical Center, Anyang 14068, Republic of Korea; honeyny78@gmail.com (N.Y.K.); kyoons@gmail.com (S.H.); 8 Department of Pathology, Hallym University Dongtan Sacred Heart Hospital, Hallym University College of Medicine, Hwaseong 18450, Republic of Korea; yhchoi@hallym.or.kr; 9 Research Institute for Complementary & Alternative Medicine, Hallym University, Chuncheon 24252, Republic of Korea; 10 Department of Pathology, Uijeongbu Eulji Medical Center, Eulji University School of Medicine, Uijeongbu 11759, Republic of Korea; kyueng@gmail.com; 11 Suseo Seoul E.N.T. Clinic and MD Analytics, Seoul 06349, Republic of Korea; mdanalytics@naver.com

**Keywords:** chronic kidney disease, Alzheimer’s disease, longitudinal study, national health screening cohort

## Abstract

Chronic kidney disease (CKD) and Alzheimer’s disease (AD) are common chronic diseases in the elderly population. Although a relationship between CKD and the occurrence of AD has been proposed, previous research results have been disputed, and further investigation is necessary to confirm this relationship. In this longitudinal follow-up study, we examined data from the Korean National Health Insurance Service-Health Screening Cohort, consisting of 15,756 individuals with CKD and 63,024 matched controls aged ≥40 years who received health check-ups between 2002 and 2019. Overlap-weighted Cox proportional hazard regression models were exploited to calculate hazard ratios (HRs) for the association between CKD and AD. During the monitoring period, individuals with CKD had a greater incidence of AD than those without CKD (15.80 versus 12.40 per 1000 person years). After accounting for various factors, CKD was significantly associated with a 1.14-fold increased likelihood of developing AD, with a 95% confidence interval ranging from 1.08 to 1.20. In subgroup analysis, this relationship persisted irrespective of age (≥70 or <70), sex, income, smoking status, alcohol consumption, place of residence, or fasting blood glucose level. Additionally, the association between CKD and AD was still evident among patients who were overweight or obese, those with normal blood pressure or cholesterol levels, and those without any other health conditions or with a CCI score of ≥2. These results suggest that CKD could increase the probability of developing AD in the Korean adult population irrespective of demographic or lifestyle conditions. This may make it challenging to predict AD in patients with CKD, emphasizing the importance of frequent AD screening and management.

## 1. Introduction

Chronic kidney disease (CKD) and Alzheimer’s dementia (AD) are common chronic diseases in the elderly population [1,2]. CKD is a major global public health concern, particularly in Korea, where it affects approximately 10–13% of the general population [1,3]. Among individuals aged ≥60, the prevalence of CKD increased to approximately 40% [4]. CKD refers to anomalies in the structure or function of the kidneys that persist for more than 3 months [5]. If left untreated, CKD can progress to the point where dialysis or kidney transplantation may be required [6]. Additionally, CKD poses a meaningful hazard for cardiovascular events and increases the risk of all-cause mortality [6]. Between the years 1990 and 2017, there was a 41.5% surge in the worldwide mortality rate associated with CKD across all age demographics [7]. AD is the predominant cause of dementia and is estimated to affect approximately 35 million individuals globally [2]. AD is characterized by a combination of protein abnormalities, specifically aberrant deposits of β-amyloid and tau proteins in the brain [8,9]. These protein deposits cause progressive neurodegenerative damage [8,9]. According to the 2020 report on dementia by the Korean Dementia Observatory, the prevalence of dementia over the last 10 years, from 2010 to 2019, has increased by 30% [2]. It is anticipated that the number of individuals over the age of 65 years that will be affected by dementia in Korea will increase to over 3 million by 2050, which is approximately 16% of the population [2]. This is a significant increase from the 10% reported in 2019 [2]. Owing to the aging population and evolving lifestyles in Korea [10], CKD and AD have emerged as significant public health concerns, posing a substantial financial and health burden on society [1,2].

Recent epidemiological studies have shown a link between CKD and a heightened risk of dementia [11,12,13] or cognitive impairment [14,15]. This association raises concerns about the hazard of comorbid dementia in elderly individuals with CKD. These results may support the theory of crosstalk between the kidneys and nervous system, as seen in both in vitro and in vivo studies [16,17]. Sustained kidney injury and reduced renal function can have detrimental effects on the function and structure of the kidneys, brain, gut, lungs, heart, and immune system [18,19]. Given that the kidneys and brain are both end organs, they share similar anatomical and vascular systems and hemodynamic features, which make them vulnerable to vascular damage [16,18,20]. Therefore, CKD and AD may share common risk factors and potential underlying mechanisms [21]. Indeed, multiple risk factors, including advanced age (>60 years), family history, lifestyle factors (poor diet, smoking, and excessive alcohol consumption), cardiovascular risk factors (hypertension, high cholesterol, and diabetes), and exposure to certain toxins are related to both CKD and AD [21,22]. In contrast, there have been various contradictory findings regarding the relationship between CKD and dementia [23,24], particularly AD [25,26]. Some studies have reported no significant association between these two conditions [24,25,26].

A meta-analysis of 10 studies found a noteworthy link between CKD and cognitive impairment [27]. However, there was a high degree of variation among the studies, and it was not possible to determine a specific association between CKD and AD onset [27]. Previous studies exploring the relationship between CKD and dementia did not differentiate between AD and other forms of dementia [12,25,28]. In some situations, AD was included in a limited number of cases [11], which limited the applicability of the findings to AD. The sample sizes of the CKD and control groups in many of these studies were often unequal in terms of demographic data and predominantly consisted of community-based cohorts [11,13,14]. Patients in the CKD group were older, had a more deviated prevalence of comorbidities such as diabetes and cardiovascular disease, and were less likely to have completed high school or earn a high income [12,13,14,15,26]. Therefore, additional validation using national population cohort data with well-matched and balanced demographics is required to minimize the impact of confounding factors [21]. As CKD and AD share common risk factors and reciprocal associations, a long-term follow-up study that considers potential mutual confounders is also required to confirm the link between CKD and the likelihood of developing AD.

As assessing the impact of CKD on dementia may obscure its effect on AD, we conducted a longitudinal follow-up study with a specific focus on the association between CKD and the likelihood of developing AD. To achieve this, we used data from the Korean national public healthcare system. We hypothesized that the impact of CKD on the likelihood of developing AD may vary based on patient factors, such as sex, age, social or economic status, and the presence of comorbid conditions. Therefore, this study aimed to explore the onset of AD and to suggest possible preventive measures for individuals with CKD.

## 2. Results

### 2.1. Baseline Characteristics

This study included 17,478 individuals with CKD who were matched for age, sex, income, and place of residence, with a comparison group of 497,388 participants. Table 1 summarizes the baseline characteristics of both groups before and after an overlap-weight-adjusted propensity score matching procedure.

### 2.2. Association of Occurrence of AD between the CKD Group and Controls

**Figure 1:**
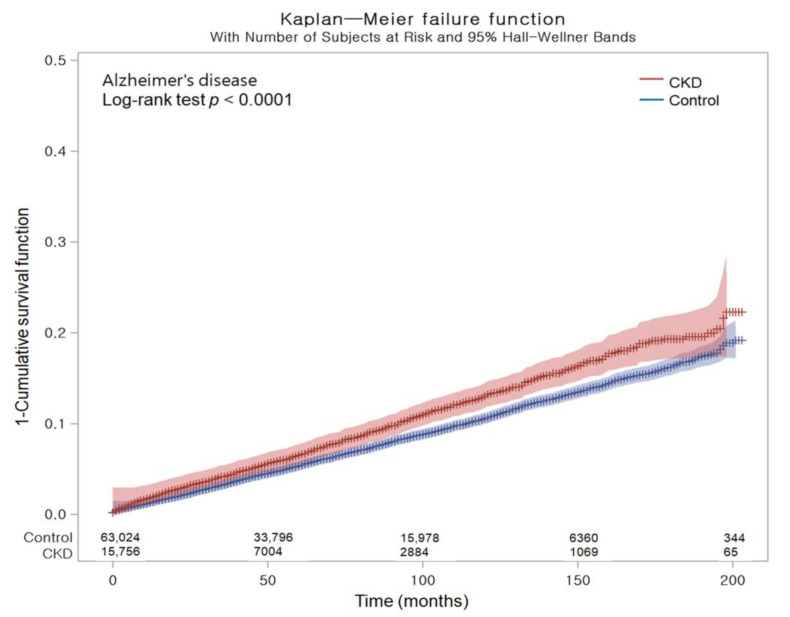
Kaplan–Meier probability of the incidence of Alzheimer’s disease in chronic kidney disease and the control populations within 16 years of the index date.

Table 2 shows the crude and adjusted hazard ratios (HRs) of CKD for incident AD. Overall, AD occurred in 1050 (6.66%) patients among the 15,756 patients with CKD and 4053 (6.43%) of the 63,024 control cohort. With their follow-up durations (66,403 and 326,315 person-years, respectively), the incidence rate of AD (15.80 per 1000 person-years) was higher in the CKD group than in the control group (12.40 per 1000 person-years). After adjusting for demographic factors and medical comorbidities, Cox regression analysis documented that individuals with CKD had a greater probability of developing AD compared to the control group (HR, 1.14; 95% confidence intervals (CIs), 1.08–1.20; *p* < 0.001). Kaplan–Meier analysis and log-rank test indicated a greater likelihood of developing AD among individuals with CKD in comparison with the control group throughout the 16-year follow-up period (*p* < 0.0001; Figure 1).

### 2.3. Subgroup Analysis

To further investigate the association between CKD and the incidence of AD, patients were stratified on the basis of sex, age, income, and residential area (Table 3). The event of AD was found to be meaningfully greater in individuals with CKD who were either <70 or ≥70 years ([HR, 1.27; 95% CI, 1.13–1.44; *p <* 0.001] and [HR, 1.10; 95% CI, 1.037–1.16; *p* = 0.004], each); male or female ([HR, 1.15; 95% CI, 1.07–1.24; *p* < 0.001] and [HR, 1.12; 95% CI, 1.04–1.21; *p* = 0.005], each); with a low or high income status ([HR, 1.17; 95% CI, 1.08–1.27; *p* < 0.001] and [HR, 1.11; 95% CI, 1.03–1.19; *p* = 0.004], each); and living in either urban or rural areas ([HR, 1.20; 95% CI, 1.10–1.31; *p* < 0.001] and [HR, 1.09; 95% CI, 1.02–1.17; *p* = 0.014], each).

Subgroup analysis revealed that CKD was significantly associated with an increased risk of developing AD, regardless of smoking history, alcohol consumption, or fasting blood glucose level. Additionally, individuals with CKD were more likely to develop AD if they were overweight (HR, 1.19; 95% CI, 1.06–1.32; *p* = 0.002) or obese (HR, 1.19; 95% CI, 1.08–1.30; *p* < 0.001), had a systolic blood pressure <140 mmHg and diastolic blood pressure <90 mmHg (1.16; 95% CI, 1.09–1.24; *p* < 0.001), a total cholesterol <200 mg/dL (HR, 1.19; 95% CI, 1.11–1.28; *p* < 0.001), CCI scores = 0 (HR, 1.20; 95% CI, 1.07–1.34; *p* = 0.001), or CCI scores ≥2 (HR, 1.11; 95% CI, 1.02–1.19; *p* = 0.010).

## 3. Discussion

This study revealed that there was a slight elevation in the risk of developing AD in Korean adults with CKD than in those without CKD over a 16-year observation period. Using a propensity score overlap-weighted Cox proportional hazard regression analysis adjusted for confounding factors such as demographics, socioeconomics, lifestyle, and comorbidities, the study found that CKD could be an independent risk factor for developing AD. Those with CKD had a 14% higher chance (95% CI, 1.08–1.20) of occurring AD than those without CKD. The study found that the effect of CKD on the prevalence of AD remained consistent regardless of various factors, such as sex, age, income, residential location, smoking history, alcohol consumption, fasting blood glucose level, and certain comorbidities, such as obesity or CCI scores ≥2. This suggests that predicting AD in patients with CKD may be challenging, and highlighting the need for regular screening of AD in these patients is of great importance.

Our results confirmed the findings of prior studies suggesting a relationship between CKD and the hazard of dementia. This is the first study that demonstrates the association between CKD and the incidence of AD. This study found a persistently increased risk of developing AD in participants with CKD throughout the 16-year follow-up period when compared with the control group. In an earlier community-based population study (*n* = 2406) in the USA, CKD was linked to a more augmented hazard of cognitive impairment, even after controlling for age, sex, race, and education [14]. This study emphasizes a positive relationship between the severity of CKD and cognitive impairment, indicating a causal relationship between CKD and cognitive impairment [14], which could not be assessed owing to the lack of data on the severity of CKD and AD in the national insurance data in the present study. However, individuals who had been diagnosed with cognitive impairment in the study were not officially diagnosed with dementia or AD by physicians [14]; only questionnaire assessments were performed by trained personnel [14]. Another community-based population study of individuals aged ≥65 years living in a typical agricultural area in northern Japan (*n* = 497) demonstrated that CKD has a 5.3-fold greater risk (95% CI, 1.7–16.2) for the incidence of dementia [11]. This remarkably high value may be explained by the fact that rural regions of the country may be disproportionally affected by dementia or AD compared to urban areas, possibly owing to underdiagnosis or risk factors [29]. However, only 60.7% (17/28) of the patients with dementia had AD in this study. Body mass index was not included as a variable in the study, and the 5-year follow-up timespan may not have been lengthy enough to fully evaluate the development of dementia [11]. A community cohort study conducted in the United Kingdom, which compiled data for over 10 years and included 306 cases of dementia, found that CKD was involved with an HR of 1.37 (95% CI 1.02–1.85) for the development of dementia [13]. The community-based cohort studies mentioned above may have limitations owing to confounding factors depending on the residence area [11,13,14]. A massive nationwide population-based study that employed the National Health Insurance Research Database in Taiwan (*n* = 37,049 for the CKD group; *n* = 74,098 for the control group), which has a health insurance plan similar to that of Korea, determined the association between CKD and the probability of subsequent dementia, with an overall HR of 1.41 (95% CI 1.32–1.50) [12]. This study is noteworthy for its in-depth investigation of comorbidities and medications as predictive factors for incident dementia. However, this study did not focus on patients with AD.

However, some studies have found conflicting evidence regarding the link between CKD and AD [26], or dementia [23,24,26]. In a prospective, population-based cohort study (*n* = 6256) in Germany, which involved over 17 years of follow-up, there was no significant involvement between CKD and an increased hazard of developing all-cause dementia (HR 0.95, 95% CI 0.69–1.29), AD (HR 0.94, 95% CI 0.55–1.63), or vascular dementia (HR 1.06, 95% CI 0.65–1.70) [26]. Previous negative studies on this issue did not accomplish the exact balance in terms of sociodemographic and health profiles at baseline between the CKD and control groups [23,24,26]. The heterogeneity in terms of demographic data differences likely caused huge differences in the original qualities of the research groups concerning the same participant [30]. To minimize potential confounding factors in our investigation, we planned a preferable research design, which involved applying nationwide organized data and overlap weighting adjustment to reduce differences between the cohorts. The conclusion drawn from our study was that there was a small but statistically meaningful increase in the HR for AD following CKD, even after adjusting for age, income, place of residence, smoking status, alcohol consumption, and fasting blood glucose levels. This finding was confirmed using a larger sample size of 15,756 individuals with CKD who were closely matched with 63,024 participants without CKD, indicating that CKD may be an independent predictor of AD. This conclusion is consistent with previous studies that investigated the link between CKD and dementia [11,13,28,31]. The fact that the effect of CKD on AD was observed consistently over a 16-year follow-up period in our study is particularly noteworthy and may be clinically significant, as previous studies on this topic had follow-up durations ranging from 4 to 17 years [13,15,24,26,28,31].

Individuals with CKD often have a more enhanced risk of occurring cardiovascular diseases such as arrhythmias, heart failure, coronary artery disease, and sudden cardiac death [6]. The influence of cardiovascular risk elements on the risk of dementia or cognitive impairment in individuals with CKD has not been investigated [13,23]. According to a recent study, stroke is not an independent risk factor for developing dementia in patients with CKD [21]. Adjustment for incident stroke modestly attenuates the risk of cognitive impairment in patients with CKD [14]. In other studies, excluding participants who had a stroke during the follow-up period did not affect the association between CKD and the probability of subsequent dementia [13,23]. One study found that the association between CKD and dementia was not influenced by the presence of small vessel disease [28]. Additionally, even after controlling for major cardiometabolic conditions, such as obesity, hypertension, diabetes, and coronary heart disease, the risk of dementia following CKD persisted [14]. This suggests that the higher hazard of dementia related to CKD may not be attributable to cardiovascular factors [13,14,23,28]. In our subgroup analyses, CKD was associated with elevated HRs in AD, regardless of cardiovascular risk factors, which corresponds with a Taiwanese study [12]. Furthermore, we noted that a subset of CKD patients without hypertension or hyperlipidemia had an increased probability of developing AD. CKD patients without hyperlipidemia had a greater incidence of dementia than those of the non-CKD group [12], which is consistent with our study. Other comorbidities or undefined risk factors may interact with CKD and further increase the risk of developing AD [12].

The mechanisms underlying the association between CKD and AD development are unclear. However, our study found that cardiovascular risk factors are unlikely to fully explain the link between CKD and AD, which is consistent with the findings of other studies on this topic [13,14,23]. Many researchers have attempted to explain the increased risk of cognitive disorders and dementia by highlighting the high prevalence of symptomatic and subclinical ischemic cerebrovascular lesions [11,12,31]. However, other mechanisms may contribute to the development of cognitive disorders and dementia, such as the direct damage to neurons caused by uremic toxins [32]. These mechanisms may be particularly relevant in cases where there is no clear cerebrovascular disease [32]. The kidney–brain axis communicates to maintain homeostasis in the body because they share closely related anatomical and physiological aspects [20]. The interplay between the kidney and brain contributes to the pathophysiology of neurological diseases and has only been recently recognized [21,32]. In this context, first, the increased risk of developing AD may result from impaired kidney function leading to the decreased clearance of toxic β-amyloid, a protein that forms the plaques found in the brains of patients with AD [21]. An animal model study found that performing a unilateral nephrectomy, which involves removing one kidney, led to an increase in the deposition of β-amyloid in the brain [33]. This was accompanied by the aggravation of β-amyloid and tau protein deposition, glial activation, neuroinflammation, and neuronal loss [33]. Additionally, cognitive deficits were also observed to worsen in the mice [33]. Individuals with CKD had higher levels of tau protein, another protein implicated in the development of AD, in their cerebrospinal fluid than those without CKD [34]. In a prospective study, CKD was associated with dementia-related blood biomarker levels of phosphorylated tau 181, which may result from reduced kidney clearance [26]. Cognitive improvement was noted in kidney transplant recipients [35]. Another potential mechanism linking CKD and AD involves the uremic toxin homocysteine [36]. Elevated plasma homocysteine levels are associated with both atrophic changes in the brain and glomerular injury [37]. This toxin may contribute to the pathogenesis of AD by activating N-methyl-D-aspartate receptors, leading to excessive calcium influx, increased oxidative stress, and inflammation within neurons, ultimately resulting in cell death [38]. Oxidative stress has also been identified as a significant element in the development of age-related neurodegenerative diseases, such as AD [39]. This, in turn, can cause changes in the histological characteristics of AD [21]. Furthermore, it is possible that the presence of certain metals, such as aluminum, copper, zinc, and iron, which cannot be properly eliminated owing to reduced kidney function, may contribute to chronic increases in oxidative stress and the release of inflammatory cytokines [40,41]. This can ultimately lead to an imbalanced concentration of neurotransmitters and promote the accumulation of β-amyloid and Na+/K+-ATPase activity, potentially contributing to AD development.

The strength and reliability of this study are rooted in the use of a representative nationwide cohort database that allows for the matching of patient and control members through overlap-weighted propensity score matching. This technique helped minimize selection bias and create study groups that were similar to those in randomized clinical trials, adding to the integrity of the study. Although previous studies have indicated a high prevalence of CKD and AD among certain groups, such as women, elderly people, those with a low income, and those living in rural areas [12,13,14,15,26], our study created a balanced distribution of demographic and health-related factors by matching 15,756 individuals with CKD to 63,024 participants without CKD. This allowed us to accurately investigate the association between CKD and AD. Through this procedure, we found that individuals with CKD were more likely to develop AD, regardless of factors such as sex, age, income, residential location, smoking history, and alcohol consumption. This suggests that the association between CKD and AD is not limited to specific subgroups. Second, the use of the Korean National Health Insurance Service-Health Screening Cohort (KNHIS-HSC) database in our study allowed a complete medical history to be obtained from every hospital and clinic throughout the country. This significantly enhanced the generalizability and accuracy of our findings. Third, another advantage of our study is that we carefully considered and adjusted for potential confounding variables. This included adjusting for socioeconomic status, such as income and area of residence, and lifestyle-related risk factors, such as alcohol consumption, blood pressure, obesity, fasting blood glucose, total cholesterol level, and smoking. We also considered comorbidities, which further enhanced the reliability and accuracy of our findings. Finally, our study also benefited from a lengthy 16-year follow-up period, which is one of the longest and most extensive longitudinal studies investigating the relationship between CKD and AD. This provided a significant advantage in terms of detecting and analyzing the potential associations between the two conditions over an extended period.

Our findings possessed some limitations. First, the observational and retrospective nature of our study design meant that we could not definitively establish a causal relationship between CKD and AD. Additionally, we did not investigate the underlying mechanisms that might explain the association between these two conditions. Second, we included only Korean citizens over the age of 40 years and relied on diagnosis codes obtained from health insurance data in Korea. This means that there may be some unmeasured confounding variables that were not accounted for, and that the results may not be generalizable to other populations. Third, the KNHIS-HSC database used in our study did not contain information regarding the severity of CKD or AD, family history, personal genetics, or dietary habits. This lack of information may have limited our ability to fully understand and investigate the relationship between CKD and AD.

## 4. Materials and Methods

### 4.1. Ethics

The ethics committee of Hallym University approved this study (2019-10-023) and written informed consent was not required by the Institutional Review Board in accordance with their guidelines and regulations. The authors used data from the KNHIS-HSC, which provides population-based electronic files for research purposes that are de-identified to protect the anonymity of the Korean population, as earlier described [42,43,44]. The diagnostic codes used in this study followed the International Classification of Diseases, 10th Revision, Clinical Modification (ICD-10-CM).

### 4.2. Exposure (Chronic Kidney Disease)

To identify participants with CKD, the researchers categorized those who had been diagnosed with CKD (ICD-10 code: N18) at least twice or those with unspecified kidney failure (ICD-10 code: N19) as CKD patients. Additionally, participants who had received regular dialysis treatment (hemodialysis and/or peritoneal dialysis) were included if they had corresponding treatment codes (O7010, O7020, and O7070).

### 4.3. Outcome (AD)

In this study, AD was defined using either the G30 code or F00 code (dementia in AD). To ensure the accuracy of the diagnosis, only participants who had been treated for AD two or more times were included in the analysis.

### 4.4. Participant Selection

**Figure 2:**
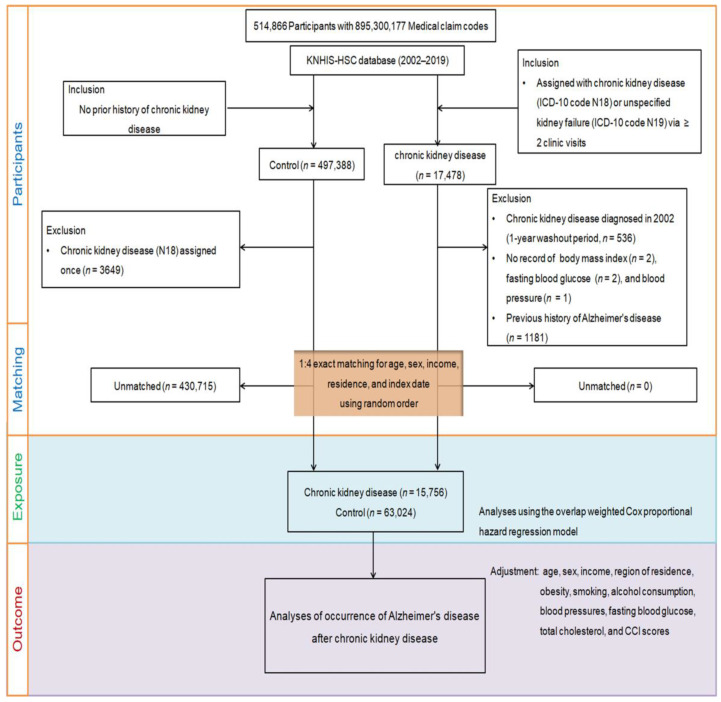
A schematic diagram of the participant selection process to analyze data from 514,866 participants. From this pool, 15,756 individuals with chronic kidney disease (CKD) were matched with 63,024 control participants based on factors such as age, sex, income, and region of residence.

From the KNHIS-HSC dataset, individuals aged ≥40 years with medical claim codes between 2002 and 2019 were included, resulting in a total of 514,866 adult patients with 895,300,177 medical claim codes. Among them, 17,478 were identified as having CKD. Individuals who were not diagnosed with CKD between 2002 and 2019 were included in the control group (*n* = 497,388). To avoid potential bias in the analysis of pre-existing AD, individuals with CKD diagnosed in 2002 were excluded from the study (*n* = 536) to allow for a 1-year washout period. Participants with CKD who did not have any recorded BMI (*n* = 2), fasting blood glucose (*n* = 2), or blood pressure (*n* = 1) values were excluded from the analysis. Participants in the control group who were diagnosed with ICD-10 code N18 were excluded from the study (*n* = 3649). The study employed a 1:4 matching strategy to create a control group that was comparable to participants with CKD in terms of age, sex, income, and region of residence. To ensure unbiased selection, the control group was randomly ordered and selected from the top of the list in a one-to-one match with participants with CKD. To ensure that both groups were assessed simultaneously, the index date for the control participants was set to match that of the corresponding participants with CKD. To ensure that the CKD and control groups were comparable, participants from the comparison group who passed away before the index date were excluded from the analysis. Furthermore, participants in the two groups with a history of AD prior to the index date were excluded to avoid any bias in the analysis. In the CKD group, a total of 1181 participants were excluded because they were left-truncated, meaning that they did not meet the inclusion criteria during the matching procedure. For the control group, 430,715 participants were excluded during the matching procedure. After excluding participants based on the aforementioned criteria, 15,756 participants with CKD were selected and matched with 63,024 participants in the control group in a ratio of 1:4. The participant selection and matching process is shown in Figure 2.

Furthermore, we searched for newly diagnosed cases of AD by identifying newly assigned ICD-10 codes for AD in both the CKD and control groups between each individual’s index date and the end of 2019.

### 4.5. Covariates

The study categorized participants into 10 age groups, with each group representing a 5-year interval, and five income groups ranging from class 1 (lowest income) to class 5 (highest income) [45]. The researchers categorized the participants’ residential areas as either urban or rural using the same methodology as in a previous study [46,47]. The study used the same categorization method as a previous study for three variables: tobacco smoking, alcohol consumption, and obesity, which was based on the participant’s body mass index in kg/m^2^ [48]. The study utilized data on several health measures, including systolic and diastolic blood pressures (mmHg), fasting blood glucose levels (mg/dL), and total cholesterol levels (mg/dL) [45]. The CCI is a commonly used tool to assess the overall burden of disease in individuals, which considers the presence of 17 different comorbid conditions. Each participant was given a score based on the severity and number of diseases using the CCI, which includes 17 comorbidities. The CCI score ranges from 0 (no comorbidities) to 29 (multiple comorbidities) [49,50]. In this study, CKD (ICD-10 codes: N18 and N19) was excluded from the CCI score. The CCI was used as a continuous variable.

### 4.6. Statistical Analyses

The research compiled proportions for categorical data and computed means along with their corresponding standard deviations for continuous data. For comparative purposes, chi-squared tests were employed for categorical variables, while *t*-tests were utilized for continuous variables. To assess the distribution of the general characteristics between the cohorts, the standardized difference was applied. To ensure that the covariates were balanced and to increase the effective sample size, propensity score overlap weighting was performed. We used multivariable logistic regression with all covariates to calculate the propensity scores. For overlap weighting, participants with CKD were weighted using the probability of the propensity score, while control participants were weighted using the probability of 1 − propensity score. The overlap weighting was calculated between 0 and 1 to achieve perfect balance and increase the precision [51,52,53]. The study used standardized differences to compare the general characteristics between the CKD and control groups before and after weighting. The accuracy of the matching groups was evaluated by comparing the absolute standardized differences of the covariates before and after matching. A value of <0.20 was considered an appropriate balance [54].

**Figure 3:**
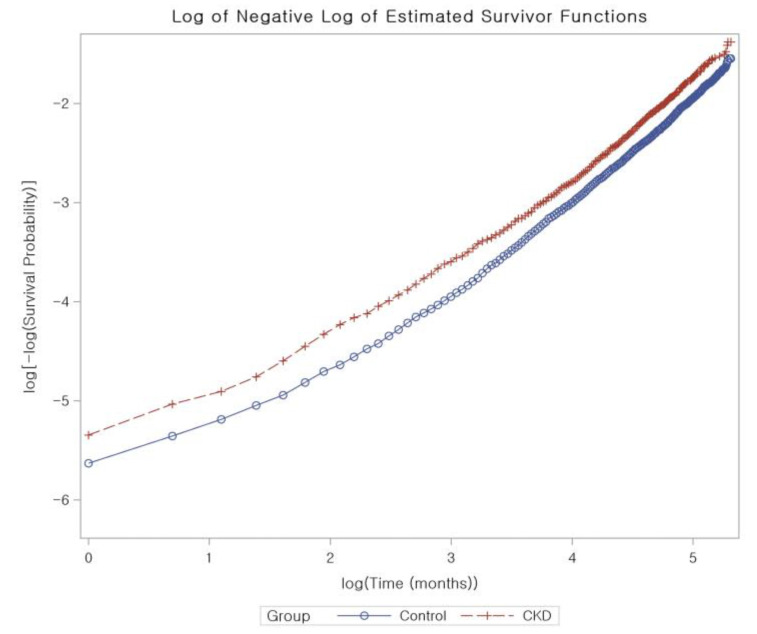
The proportional hazard assumptions for chronic kidney disease using log-minus-log plots. The analysis does not identify any violations of these assumptions, indicating that our results are reliable and accurate.

We calculated the crude incidence rates and incidence rate difference by dividing the number of participants who experienced a specific event by the total person years of follow-up, illustrated as cases per 1000 person years. Kaplan–Meier analysis and log-rank tests were used to compare the cumulative probability of incident AD in the CKD group with that of the control group. To adjust for potential confounders and to estimate the overlap-weighted HRs and 95% CIs of CKD for incident AD, we used Cox proportional hazard regressions with an overlap weight for both unadjusted and overlap-weighted (adjusted for all covariates) models. To ensure the validity of the results, we confirmed that the proportional hazard assumptions were met by creating log-minus-log plots, and found no violations of these assumptions (Figure 3).

## 5. Conclusions

Our study suggests that Korean adults with CKD may have a slightly increased risk of developing AD regardless of their demographic or lifestyle characteristics. This population-based nationwide study cautiously suggests a potential association between CKD and incident AD in Korean adults. However, further research is needed to confirm this link and investigate the underlying mechanisms in greater detail.

## Figures and Tables

**Table 1:** General Characteristics of Participants.

Characteristics	Before Overlap Weighting Adjustment			After Overlap Weighting Adjustment				
	CKD	Control	Standardized Difference	CKD	Control	Standardized Difference	*t* or χ^2^ Value ^1^	*p*-Value
Age (*n*, %)			0.00			0.00	0.00	1.000
40–44	98 (0.62)	392 (0.62)		75 (0.64)	75 (0.64)			
45–49	362 (2.30)	1448 (2.30)		261 (2.24)	261 (2.24)			
50–54	950 (6.03)	3800 (6.03)		688 (5.90)	688 (5.90)			
55–59	1861 (11.81)	7444 (11.81)		1359 (11.66)	1359 (11.66)			
60–64	2288 (14.52)	9152 (14.52)		1666 (14.29)	1666 (14.29)			
65–69	2567 (16.29)	10,268 (16.29)		1883 (16.15)	1883 (16.15)			
70–74	2804 (17.80)	11,216 (17.80)		2089 (17.92)	2089 (17.92)			
75–79	2587 (16.42)	10,348 (16.42)		1943 (16.67)	1943 (16.67)			
80–84	1579 (10.02)	6316 (10.02)		1187 (10.18)	1187 (10.18)			
85+	660 (4.19)	2640 (4.19)		507 (4.35)	507 (4.35)			
Sex (*n*, %)			0.00					
Male	10,548 (66.95)	42,192 (66.95)		7816 (67.04)	7816 (67.04)			
Female	5208 (33.05)	20,832 (33.05)		3842 (32.96)	3842 (32.96)			
Income (*n*, %)			0.00			0.00	0.00	1.000
1 (lowest)	2717 (17.24)	10,868 (17.24)		2000 (17.16)	2000 (17.16)			
2	1831 (11.62)	7324 (11.62)		1357 (11.64)	1357 (11.64)			
3	2272 (14.42)	9088 (14.42)		1678 (14.39)	1678 (14.39)			
4	3176 (20.16)	12,704 (20.16)		2340 (20.08)	2340 (20.08)			
5 (highest)	5760 (36.56)	23,040 (36.56)		4283 (36.74)	4283 (36.74)			
Region of residence (*n*, %)			0.00			0.00	0.00	1.000
Urban	6820 (43.29)	27,280 (43.29)		5046 (43.28)	5046 (43.28)			
Rural	8936 (56.71)	35,744 (56.71)		6612 (56.72)	6612 (56.72)			
Obesity ^†^ (*n*, %)			0.16			0.00	0.00	1.000
Underweight	395 (2.51)	1971 (3.13)		307 (2.63)	307 (2.63)			
Normal	4817 (30.57)	22,314 (35.41)		3674 (31.51)	3674 (31.51)			
Overweight	4154 (26.36)	17,210 (27.31)		3109 (26.67)	3109 (26.67)			
Obese I	5678 (36.04)	19,850 (31.50)		4110 (35.25)	4110 (35.25)			
Obese II	712 (4.52)	1679 (2.66)		458 (3.93)	458 (3.93)			
Smoking status (*n*, %)			0.02			0.00	0.00	1.000
Nonsmoker	9946 (63.13)	40,439 (64.16)		7394 (63.42)	7394 (63.42)			
Past smoker	1693 (10.75)	6674 (10.59)		1258 (10.79)	1258 (10.79)			
Current smoker	4117 (26.13)	15,911 (25.25)		3006 (25.79)	3006 (25.79)			
Alcohol consumption (*n*, %)			0.07			0.00	0.00	1.000
<1 time a week	11,357 (72.08)	43,333 (68.76)		8299 (71.18)	8299 (71.18)			
≥1 time a week	4399 (27.92)	19,691 (31.24)		3360 (28.82)	3360 (28.82)			
SBP (Mean, SD)	131.85 (18.41)	128.67 (16.26)	0.18	130.87 (15.42)	130.87 (7.30)	0.00	0.00	1.000
DBP (Mean, SD)	78.86 (11.56)	78.14 (10.33)	0.07	78.62 (9.83)	78.62 (4.54)	0.00	0.00	1.000
Fasting blood glucose (Mean, SD)	115.36 (48.85)	103.60 (28.08)	0.30	109.75 (32.48)	109.75 (16.57)	0.00	0.00	1.000
Total cholesterol (Mean, SD)	190.49 (45.68)	193.55 (38.73)	0.07	190.88 (38.95)	190.88 (16.78)	0.00	0.00	1.000
CCI score (Mean, SD)	2.13 (2.19)	1.07 (1.69)	0.54	1.77 (1.66)	1.77 (0.97)	0.00	0.00	1.000
AD (*n*, %)	1050 (6.66)	4053 (6.43)	0.01	712 (6.11)	894 (7.67)	0.06	21.99	<0.001

Abbreviations: CCI—Charlson Comorbidity Index; SBP—Systolic blood pressure; DBP—Diastolic blood pressure; Alzheimer dementia—AD; ^†^ Obesity (BMI, body mass index, kg/m^2^) was categorized as <18.5 (underweight), ≥18.5 to <23 (normal), ≥23 to <25 (overweight), ≥25 to <30 (obese I), and ≥30 (obese II). Categorical variables were compared using chi-squared tests and continuous variables were compared using *t*-tests; ^1^ The chi-squared test or independent *t*-test were analyzed, where significance was at *p* < 0.05.

**Table 2:** Crude and overlap propensity score weighted hazard ratios (95% confidence interval) of CKD for AD with subgroup analyses according to age, sex, income, and region of residence.

	*n* of Event/*n* of Total (%)	Follow-Up Duration (PY)	IR per 1000 (PY)	IRD (95% CI)	Hazard Ratios for AD			
					Crude	*p*	Overlap Weighted Model ^†^	*p*
Total participants								
CKD	1050/15,756 (6.66)	66,403	15.80	3.40 (2.44–4.34)	1.25 (1.17–1.34)	<0.001 *	1.14 (1.08–1.2)	<0.001 *
Control	4053/63,024 (6.43)	326,315	12.40		1		1	
Age < 70 years old								
CKD	244/8126 (3.00)	45,015	5.42	2.12 (1.51–2.74)	1.72 (1.48–1.99)	<0.001 *	1.27 (1.13–1.44)	<0.001 *
Control	707/32,504 (2.18)	214,462	3.30		1		1	
Age ≥ 70 years old								
CKD	806/7630 (10.56)	21,388	37.70	7.80 (5.19–10.35)	1.25 (1.16–1.35)	<0.001 *	1.10 (1.03–1.16)	0.004 *
Control	3346/30,520 (10.96)	111,853	29.90		1		1	
Male								
CKD	566/10,548 (5.37)	43,453	13.00	3.00 (1.94–4.06)	1.28 (1.16–1.40)	<0.001 *	1.15 (1.07–1.24)	<0.001 *
Control	2140/42,192 (5.07)	213,445	10.00		1		1	
Female								
CKD	484/5208 (9.29)	22,950	21.10	4.20 (2.26–6.03)	1.22 (1.10–1.35)	<0.001 *	1.12 (1.04–1.21)	0.005 *
Control	1913/20,832 (9.18)	112,870	16.90		1		1	
Low income group								
CKD	456/6820 (6.69)	28,399	16.10	3.60 (2.09–5.01)	1.27 (1.15–1.41)	<0.001 *	1.17 (1.08–1.27)	<0.001 *
Control	1780/27,280 (6.52)	142,343	12.50		1		1	
High income group								
CKD	594 /8936 (6.65)	38,004	15.60	3.20 (2.02–4.53)	1.24 (1.13–1.35)	<0.001 *	1.11 (1.03–1.19)	0.004 *
Control	2273/35,744 (6.36)	183,972	12.40		1		1	
Urban resident								
CKD	442/6820 (6.48)	30,567	14.50	4.10 (2.72–5.32)	1.37 (1.23–1.52)	<0.001 *	1.20 (1.10–1.31)	<0.001 *
Control	1535/27,280 (5.63)	147,086	10.40		1		1	
Rural resident								
CKD	608/8936 (6.80)	35,836	17.00	3.00 (1.55–4.28)	1.18 (1.08–1.29)	<0.001 *	1.09 (1.02–1.17)	0.014 *
Control	2518/35,744 (7.04)	179,229	14.00		1		1	

Abbreviation: CKD—Chronic kidney disease; AD—Alzheimer dementia; IR—incidence rate; CI—confidence interval; IRD—incidence rate difference; PY—person- year; * Significance at *p* < 0.05; ^†^ Adjusted for age, sex, income, region of residence, obesity, smoking, alcohol consumption, systolic blood pressure, diastolic blood pressure, fasting blood glucose, total cholesterol, and Charlson Comorbidity Index scores.

**Table 3:** Subgroup analyses of crude and overlap propensity score weighted hazard ratios (95% confidence interval) of CKD for AD.

	*n* of Event/ *n* of Total (%)	Follow-Up Duration (PY)	IR per 1000 (PY)	IRD (95% CI)	Hazard Ratios for AD			
					Crude	*p*	Overlap Weighted Model ^†^	*p*
Underweight								
CKD	25/395 (6.33)	1244	20.10	−4.90 (−14.20 to 4.36)	0.76 (0.50–1.15)	0.197	0.91 (0.68–1.23)	0.547
Control	218/1971 (11.06)	8715	25.00		1		1	
Normal weight								
CKD	348/4817 (7.22)	19,540	17.80	2.80 (0.95 to 4.71)	1.16 (1.03–1.30)	0.014 *	1.08 (0.99–1.18)	0.087
Control	1708/22,314 (7.65)	114,010	15.00		1		1	
Overweight								
CKD	276/4154 (6.64)	18,487	14.90	4.30 (2.62 to 5.99)	1.39 (1.22–1.59)	<0.001 *	1.19 (1.06–1.32)	0.002 *
Control	962/17,210 (5.59)	90,563	10.60		1		1	
Obese								
CKD	401/6390 (6.28)	27,132	14.80	4.50 (3.07 to 5.87)	1.42 (1.27–1.59)	<0.001 *	1.19 (1.08–1.30)	<0.001 *
Control	1165/21,529 (5.41)	113,027	10.30		1		1	
Non-smoker								
CKD	752/9946 (7.56)	43,502	17.30	3.40 (2.12 to 4.60)	1.22 (1.13–1.32)	<0.001 *	1.14 (1.07–1.21)	<0.001 *
Control	2963/40,439 (7.33)	212,727	13.90		1		1	
Past and current smoker								
CKD	298/5810 (5.13)	22,901	13.00	3.40 (1.98 to 4.85)	1.32 (1.16–1.50)	<0.001 *	1.13 (1.02–1.26)	0.020 *
Control	1090/22,585 (4.83)	113,588	9.60		1		1	
Alcohol consumption <1 time a week								
CKD	842/11,357 (7.41)	48,781	17.30	3.60 (2.34 to 4.69)	1.23 (1.14–1.33)	<0.001 *	1.14 (1.07–1.21)	<0.001 *
Control	3108/43,333 (7.17)	226,064	13.70		1		1	
Alcohol consumption ≥1 time a week								
CKD	208/4399 (4.73)	17,622	11.80	2.37 (0.79 to 3.96)	1.23 (1.06–1.43)	0.008 *	1.13 (1.01–1.28)	0.035 *
Control	945/19,691 (4.80)	100,251	9.43		1		1	
SBP < 140 mmHg and DBP < 90 mmHg								
CKD	642/10,306 (6.23)	41,881	15.30	3.90 (2.76 to 5.04)	1.31 (1.20–1.43)	<0.001 *	1.16 (1.09–1.24)	<0.001 *
Control	2661/46,067 (5.78)	232,910	11.40		1		1	
SBP ≥ 140 mmHg or DBP ≥ 90 mmHg								
CKD	408/5450 (7.49)	24,522	16.60	1.70 (0.00 to 3.47)	1.10 (0.98–1.23)	0.091	1.09 (0.99–1.20)	0.074
Control	1392/16,957 (8.21)	93,405	14.90		1		1	
Fasting blood glucose < 100 mg/dL								
CKD	484/7405 (6.54)	33,911	14.30	2.60 (1.36 to 3.89)	1.20 (1.09–1.33)	<0.001 *	1.16 (1.07–1.25)	<0.001 *
Control	2279/34,901 (6.53)	195,609	11.70		1		1	
Fasting blood glucose ≥ 100 mg/dL								
CKD	566/8351 (6.78)	32,492	17.40	3.80 (2.39 to 5.30)	1.26 (1.15–1.39)	<0.001 *	1.12 (1.03–1.21)	0.007 *
Control	1774/28,123 (6.31)	130,706	13.60		1		1	
Total cholesterol < 200 mg/dL								
CKD	647/9734 (6.65)	38,036	17.00	4.50 (3.19 to 5.74)	1.33 (1.21–1.45)	<0.001 *	1.19 (1.11–1.28)	<0.001 *
Control	2261/36,777 (6.15)	180,229	12.50		1		1	
Total cholesterol ≥ 200 mg/dL								
CKD	403/6022 (6.69)	28,367	14.20	1.90 (0.51 to 3.37)	1.14 (1.03–1.27)	0.016 *	1.07 (0.98–1.16)	0.111
Control	1792/26,247 (6.83)	146,086	12.30		1		1	
CCI scores = 0								
CKD	159/4810 (3.31)	22,607	7.03	0.31 (−0.82 to 1.45)	1.04 (0.88–1.22)	0.67	1.20 (1.07–1.34)	0.001 *
Control	1261/34,942 (3.61)	187,732	6.72		1		1	
CCI scores = 1								
CKD	83/2695 (3.08)	10,818	7.67	−2.53 (−4.51 to −0.46)	0.74 (0.59–0.93)	0.009 *	0.95 (0.80–1.12)	0.533
Control	600/11,574 (5.18)	59,071	10.20		1		1	
CCI scores ≥ 2								
CKD	808/8251 (9.79)	32,978	24.50	−3.10 (−5.16 to −0.97)	0.87 (0.81–0.95)	0.001 *	1.11 (1.02–1.19)	0.010 *
Control	2192/16,508 (13.28)	79,512	27.60		1		1	

Abbreviation: CKD—Chronic kidney disease; AD—Alzheimer dementia; IR—incidence rate; CI—confidence interval; IRD—incidence rate difference; PY—person year; * Significance at *p* < 0.05; ^†^ Adjusted for age, sex, income, region of residence, obesity, smoking, alcohol consumption, systolic blood pressure, diastolic blood pressure, fasting blood glucose, total cholesterol, and Charlson Comorbidity Index scores.

## Data Availability

All data are available from the database of National Health Insurance Sharing Service (NHISS) https://nhiss.nhis.or.kr/ (accessed on 1 May 2022). NHISS allows access to all of this data for the any researcher who promises to follow the research ethics at some processing charge. If you want to access the data of this article, you can download it from the website after promising to follow the research ethics.
